# MHCA with SACP versus DHCA in Pediatric Aortic Arch Surgery: A Comparative Study

**DOI:** 10.1038/s41598-020-61428-x

**Published:** 2020-03-10

**Authors:** Ling Xie, Yan Xu, Guijin Huang, Mao Ye, Xiao Hu, Shiyu Shu, Harness Lynn

**Affiliations:** 1 Department of Anesthesiology, Ministry of Education Key Laboratory of Child Development and Disorders; National Clinical Research Center for Child Health and Disorders; China International Science and Technology Cooperation base of Child development and Critical Disorders; Children’s Hospital of Chongqing Medical University, Chongqing, P.R. China; 2 Department of Anesthesiology, Eye & ENT Hospital of Fudan University, Shanghai, China; 3 Division of Cardiac Surgery, Johns Hopkins Hospital, School of Medicine, Baltimore, Maryland USA

**Keywords:** Cardiology, Health care

## Abstract

The safety and efficacy of selective antegrade cerebral perfusion (SACP) in children undergoing aortic arch surgery are unclear. In this retrospective analysis, we compared moderate hypothermic circulatory arrest (MHCA; n = 61) plus SACP vs deep hypothermic circulatory arrest (DHCA; n = 53) in children undergoing aortic arch surgery during a period from January 2008 to December 2017. Demographic characteristics and the underlying anomalies were comparable between the two groups. The MHCA + SACP group had shorter cardiopulmonary bypass (CPB) time (146.9 ± 40.6 vs 189.6 ± 41.2 min for DHCA; p < 0.05) and higher nasopharyngeal temperature (26.0 ± 2.1 vs 18.9 ± 1.6 °C; p < 0.01). The MHCA + SACP group had lower rate of neurologic complications (3/61 vs 10/53 for DHCA; p < 0.05) but not complications in other organ systems. The MHCA + SACP group also had less 24-hour chest drainage (median, interquartile rage: 28.9, 12.6–150.0 vs 47.4, 15.2–145.0 ml/kg for DHCA; p < 0.05), shorter duration of postoperative mechanical ventilation (35.0, 15.4–80.3 vs 94.0, 42.0–144.0 h; p < 0.01), and shorter stay in intensive care unit (3.9, 3.0–7.0 vs 7.7, 5.0–15.0 d; p < 0.05). In regression analysis, in-hospital mortality was associated with longer CPB time. In conclusion, MHCA + SACP is associated with better short-term outcomes in children receiving aortic arch surgery under CPB.

## Introduction

Coarctation of the aorta (CoA) and interrupted aortic arch (IAA) are the most common congenital large vascular malformations^1^. Surgery should be performed as early as possible. Minimally invasive surgery is recommended for simple CoA/IAA. Patients with other comorbid abnormalities, however, may require open surgery under cardiopulmonary bypass (CPB). Deep hypothermic circulatory arrest (DHCA) is routinely used in aortic arch surgeries that require CPB to minimize cerebral metabolism and to provide clean surgical field^2^, but may lead to hypothermia-induced coagulopathy, capillary leak syndrome, microvasculature endothelial dysfunction, elevated systemic inflammatory response and multiple organ dysfunctions^3^. Meanwhile, the time of DHCA is limited by the temperature. Selective antegrade cerebral perfusion (SACP) is designed to supply regional cerebral perfusion through the circle of Willis, and allow longer CPB time than DHCA^4,5^.

More recently, SACP has been increasingly used with mild hypothermia or normothermia in aortic arch surgery^6^. Several studies suggested that SACP in combination with mild hypothermia (core temperature at 30 °C) could provide sufficient cerebral protection in aortic arch surgery that typically lasted for at least 90 minutes^7,8^. However, SACP at higher core temperatures has been associated with spinal cord injury and even cerebral thrombosis in some cases. As a result, the use of SACP with normothermia in young children remains controversial^9^.

In the current retrospective analysis, we used relatively larger number of cases to compare moderate hypothermic circulatory arrest (MHCA) in combination with SACP versus DHCA alone in children undergoing CoA/IAA surgery.

## Results

### Collection of patient data

A total of 121 pediatric cases were screened. Prior to surgery, all subjects received color Doppler echocardiography, chest computerized tomography (CT) or magnetic resonance imaging (MRI) in addition to vascular imaging. Seven cases were excluded from the analysis due to the following reasons prior to surgery: abnormal nervous system function (n = 3), renal insufficiency (n = 2) and coagulopathy (n = 2). The final analysis included 114 cases. The primary diagnoses included preductal CoA (n = 81), postductal CoA (n = 9), IAA type A (n = 12), IAA type B (n = 11), and IAA type C (n = 1). Surgery was conducted under DHCA in 53 cases and under MHCA + SACP in the remaining 61 cases.

### Preoperative patient characteristics

The two groups did not differ significantly in demographics (age and sex) and preoperative characteristics, including body weight, arch pathology (CoA/IAA), ASA grade (III/IV), ejection fraction, and renal function (Table 1). Anomalies in addition to CoA/IAA included ventricular septal defect (VSD), atrial septal defect (ASD), patent ductus arteriosus (PDA), patent foramen ovale (PFO), complete atrioventricular canal defect (CAVCD), double outlet right ventricle (DORV), total anomalous pulmonary venous connection (TAPVC), and aorta pulmonary window (APW), are listed in Table 2; there were no significant differences between the two groups.

**Table 1:** Demographics and basic clinical features.

	DHCA (n = 53)	MHCA + SACP (n = 61)	p value
Age (day), median (range)	117 (2~335)	136 (3~372)	0.516
Body weight (kg), median (range)	5.3 (2.2~11.0)	5.3 (3.6~12.4)	0.654
Male sex, n (%)	31 (58)	40 (64)	0.436
Arch pathology			0.458
CoA; n (%)	39 (73.6)	41 (67.2)	
IAA; n (%)	14 (26.4)	20 (32.8)	
ASA grade			0.997
III; n (%)	33 (62.3)	38 (62.3)	
IV; n (%)	20 (37.7)	23 (37.7)	
Preoperative ejection fraction (%)	66.8 ± 3.5	62.9 ± 4.2	0.797
Preoperative serum creatinine (μmol/L)	35.3 ± 8.5	27.9 ± 9.1	0.267
Preoperative blood urea nitrogen (mmol/L)	3.6 ± 1.3	3.9 ± 1.5	0.560

ASA = American Society of Anesthesiologists; CoA = coarctation of the aorta; IAA = interrupted aortic arch; DHCA = deep hypothermic circulatory arrest; MHCA = moderate hypothermic circulatory arrest; SACP = selective antegrade cerebral perfusion.

**Table 2:** Anomalies in addition to CoA/IAA: comparison between the DHCA and MHCA + SACP groups.

	DHCA (n = 53)	MHCA + SACP (n = 61)	p value
VSD	35	41	0.894
ASD	13	16	0.835
PDA	38	35	0.112
PFO	22	25	0.955
CAVCD	14	22	0.269
DORV	6	4	0.510
TAPVC	1	1	1.000
APW	1	2	1.000

VSD = ventricular septal defect; ASD = atrial septal defect; PDA = patent ductus arteriosus; PFO = patent foramen ovale; CAVCD = complete atrioventricular canal defect; DORV = double outlet right ventricle; TAPVC = total anomalous pulmonary venous connection; APW = aorta pulmonary window.

### Intraoperative data

The MHCA + SACP group had shorter CPB time (146.9 ± 40.6 vs 189.6 ± 41.2 min for DHCA; p < 0.05, Table 3). The lowest nasopharyngeal temperature and lowest rectal temperature were both higher in the MHCA + SACP group (26.0 ± 2.1 °C and 25.7 ± 2.6 °C) than in the DHCA group (18.9 ± 1.6 °C; 18.8 ± 1.7 °C) (p < 0.01 for both comparisons). There were no significant differences in aortic cross-clamp (ACC) time, DHCA/SACP time, and urine output during CPB.

**Table 3:** Intra- and post-operative variables: comparison between the DHCA and MHCA + SACP groups.

	DHCA (n = 53)	MHCA + SACP (n = 61)	p value
*Intraoperative*			
**CPB time (min)**	**189.6** ± **41.2**	**146.9** ± **40.6**	**0.033**
ACC time (min)	88.9 ± 19.6	75.9 ± 22.7	0.245
DHCA/SACP time (min)	32.2 ± 5.6	34.5 ± 4.2	0.321
**Lowest nasopharyngeal temperature (°C)**	**18.9** ± **1.6**	**26.0** ± **2.1**	**<0.001**
**Lowest rectal temperature (°C)**	**18.8** ± **1.7**	**25.7** ± **2.6**	**<0.001**
Urine output during bypass (ml/kg/h)	7.8 (2.3–30.0)	7.2 (2.9–80.0)	0.817
*Postoperative*			
24-hour urine output (ml/kg)	80.6 (48.7–596.0)	100.6 (51.2–454.0)	0.056
**24-hour chest drainage (ml/kg)**	**47.4 (15.2**–**145.0)**	**28.9 (12.6**–**150.0)**	**0.014**
Inotropic score (points)^a^	17.2 ± 4.8	16.7 ± 4.3	0.871
Consciousness recovery time	3.8 ± 1.5	3.4 ± 1.2	0.342
**Mechanical ventilation time (h)**	**94.0 (42.0**–**144.0)**	**35.0 (15.4**–**80.3)**	**0.008**
**ICU stay (days)**	**7.7 (5.0**–**15.0)**	**3.9 (3.0**–**7.0)**	**0.012**

DHCA = deep hypothermic circulatory arrest; MHCA = moderate hypothermic circulatory arrest; SACP = selective antegrade cerebral perfusion; CPB = cardiopulmonary bypass; ACC = aortic cross-clamp; ICU = intensive care unit;

Notes: ^a^Inotropic score was calculated as: dopamine [μg/kg/min] + dobutamine [μg/kg/min] + epinephrine [μg/kg/min] × 100 + norepinephrine [μg/kg/min] × 100 + milrinone [μg/kg/min] × 10 + vasopressin (U/kg/min) × 10000.

### Postoperative data

The MHCA + SACP group had less 24-hour chest drainage (median, interquartile rage: 28.9, 12.6–150.0 vs 47.4, 15.2–145.0 ml/kg for DHCA; p < 0.05), shorter duration of mechanical ventilation (35.0, 15.4–80.3 vs 94.0, 42.0–144.0 h; p < 0.01), and shorter stay in intensive care unit (3.9, 3.0–7.0 vs 7.7, 5.0–15.0 d; p < 0.05) (Table 3). The two groups did not differ significantly in 24-hour urine output, inotropic score^10^, and time to regain consciousness after surgery.

### Postoperative complications

The MHCA + SACP group had lower rate of nervous system complications (3/61 vs 10/53 for DHCA; p < 0.05) (Table 4). In the DHCA group, nervous system complications included delayed recovery of consciousness (n = 2), frequent episodes of convulsion (n = 3), coma (n = 1), thermal sensory disorder (n = 1), and neuroimaging findings of encephaledema (n = 2) and cerebral embolism (n = 1). The patient who developed coma after surgery died on the 25th postoperative day due to hypoxic-ischemic encephalopathy. Nervous system complications in the MHCA + SACP group included self-limiting convulsion (n = 2; 1 and 4 episodes, respectively) and neuroimaging findings of encephaledema (n = 1). No spinal cord injury was observed in either group.

**Table 4:** Postoperative complications in the DHCA vs. MHCA + SACP group.

	DHCA (n = 53)	MHCA + SACP (n = 61)	*P* Value
**Nervous system complications, n (%)**	**10 (18.9)**	**3(4.9)**	**0.019**
Delayed recovery of consciousness	2	0	
Frequent episodes of convulsion	3	2	
Coma	1	0	
Thermal sensory disorder	1	0	
Neuroimaging changes (CT or MRI)	3	1	
*Encephaledema*	2	1	
*Cerebral thrombosis*	1	0	
Spinal cord injury	0	0	
**Acute renal failure, n (%)**	1 (1.9)	2 (3.3)	1.000
**Low cardiac output syndrome, n (%)**	4 (7.5)	7 (11.5)	0.479
**Acute respiratory distress syndrome, n (%)**	4 (7.5)	6 (9.8)	0.749
**Death, n (%)**	4 (7.5)	6 (9.8)	0.749

DHCA = deep hypothermic circulatory arrest; MHCA = moderate hypothermic circulatory arrest; SACP = selective antegrade cerebral perfusion.

The two groups did not significantly differ in acute renal failure (2/61 for MHCA + SACP vs 1/53 for DHCA), low cardiac output syndrome (7/61 for MHCA + SACP vs 4/53 for DHCA) or acute respiratory distress syndrome (6/61 for MHCA + SACP vs 4/53 for DHCA) (Table 4). In-hospital mortality did not differ significantly between the two groups (9.8% for MHCA + SACP vs 7.5% for DHCA) (Table 4).

### Risk factors of in-hospital mortality

In comparison to the children who survived, children who died during hospitalization were significantly younger (median, interquartile range: 53, 27–115 vs 111, 2–374 d), lower body weight (4.1 ± 0.6 vs 6.2 ± 0.8 kg), longer CPB time (249.1 ± 99.2 vs 161.6 ± 64.8 min), and longer ACC time (126.2 ± 78.5 vs 88.0 ± 25.4 min) (p < 0.05 for all; Table 5). In multivariate logistic regression analysis, CPB time was the only independent risk factor for in-hospital mortality (Table 6).

**Table 5:** Comparison of the non-surviving vs surviving group.

	Non-survivor (n = 10)	Survivor (n = 104)	p-value
**Age (days), median (IQR)**	**53 (27**–**115)**	**111 (2**–**374)**	**0.015**
**Body weight (kg)**	**4.1** ± **0.6**	**6.2** ± **0.8**	**0.016**
Male sex, n (%)	6 (60.0)	65 (62.5)	0.260
Preoperative ejection fraction (%)	67.0 ± 11.18	66.7 ± 9.1	1.000
Preoperative serum creatinine (μmol/L), median (IQR)	30.2 (22.0–40.0)	28.5(18.1–46.9)	0.789
Preoperative blood urea nitrogen (mmol/L), median (IQR)	3.5 (2.5–4.9)	3.6 (2.1–6.45)	0.912
**CPB time (min)**	**249.1** ± **99.2**	**161.6** ± **64.8**	**0.021**
**ACC time (min)**	**126.2** ± **78.5**	**88.0** ± **25.4**	**0.034**
DHCA/SACP time (min)	37.7 ± 10.56	33.7 ± 10.7	0.657
Lowest nasopharyngeal temperature (°C)	20.89 ± 2.18	21.2 ± 3.3	0.681
Lowest rectal temperature (°C)	23.06 ± 1.97	23.4 ± 3.2	0.833
Arch pathology			1.000
CoA	7	73	
IAA	3	31	
ASA class			0.499
III	5	66	
IV	5	38	
Circulation method			0.749
DHCA	4	49	
MHCA + SACP	6	55	

ACC = aortic cross-clamp; ASA = American Society of Anesthesiologists; CoA = coarctation of the aorta; CPB = cardiopulmonary bypass; DHCA = deep hypothermic circulatory arrest; IAA = interrupted aortic arch; IQR = interquartile range; SACP = selective antegrade cerebral perfusion.

**Table 6:** Univariate analysis of risk factors for in-hospital mortality.

	p value
Age (days)	0.833
Body weight (kg)	0.645
Gender (Male)	0.334
**CPB time (min)**	**0.015**
ACC time (min)	0.776

CPB = cardiopulmonary bypass; ACC = aortic cross-clamp.

## Discussion

DHCA and MHCA + SACP are both promising techniques for cerebral protection and are widely used in pediatric aortic surgery and complex congenital heart surgery^11^. Each method has its own advantages and disadvantages. SACP provides cerebral perfusion during cardiac arrest. However, many pediatric cardiac surgeons continue to use traditional DHCA alone to carry out aortic surgery for a variety of reasons. First, DHCA uses profound cooling and complete circulation cessation, thus allowing more adequate exposure and correction of complex anomalies due to the clean surgical field. Also, children have higher anaerobic glycolytic capacity and glycogen reserves in the immature myocardium and thus higher degree of tolerance to ischemia than adults^12^. The reported increase in the risk of cerebral embolism and spinal cord injury represents an additional concern^13–15^. Last but not least, the protective function of SACP has not been confirmed in all studies. For example, Gunn *et al*. reported that 33% of patients who received arch reconstruction with SACP between 18 °C and 25 °C suffered perioperative convulsions^16^.

In the current study, the MHCA + SACP group had lower rate of neurological complications, less 24-hour chest drainage, shorter duration of mechanical ventilation and shorter ICU stay. In our opinion, the observed benefits could be partially attributed to shorter CPB time and higher core temperature in the MHCA + SACP group.

With increasing duration of cardiac arrest, the incidence of postoperative neurologic complications and mortality increases significantly^17^. Based on a recent clinical study, circulatory arrest longer than 40 minutes may lead to adverse impact on neurological outcomes^18^. The rate of neurological complications in the MHCA + SACP group decreased, despite of longer duration of cardiac arrest in the MHCA + SACP (59 min) than in the DHCA group (49 min). This finding is consistent with a study by Kornilov^19^ in infants receiving aortic arch reconstruction, and supports the neuroprotective effects of MHCA + SACP.

It is recognized that intraoperative and postoperative bleeding has a strong influence on patient prognosis^20^. The coagulation system may be significantly disordered in response to the poor physiologic state of DHCA, and this further increases the occurrence of postoperative bleeding in pediatric patients. Postoperative coagulation dysfunction is more common in young patients who have low body weights and complicated congenital heart diseases, and results in higher morbidity and mortality among these patients compared to other groups. In our study, use of MHCA + SACP significantly decreased damage to the coagulation system induced by hypothermia and CPB, as evidenced by significantly lower chest drainage volume in the MHCA + SACP group (MHCA + SACP: 28.9 ml/kg; DHCA: 47.4 ml/kg). The lowest core temperature in the MHCA + SACP group was almost 8 degrees higher than in the DHCA group.

Damage to the lungs by CPB is profound due to systemic inflammatory responses^21^ and ischemia/reperfusion^22^. In addition, CPB could cause transient pulmonary dysfunctions that in turn further decrease postoperative pulmonary compliance, increase the alveolar-arterial oxygen gradient (A-aDO_2_), damage the pulmonary alveoli and cause pulmonary atelectasis. In the current study, the duration of mechanical ventilation and the duration of ICU stay were significantly shorter in the MHCA + SACP group than in the DHCA group. The basis of the findings is unknown, but we speculate that SACP could also provide continuous oxygenated blood to the lungs through the basilar and anterior spinal arteries during CPB.

In-hospital mortality did not differ between the two groups. Also, MHCA + SACP was not associated with in-hospital mortality in the regression analysis. Similar findings were reported by Kazui *et al*.^23^ and Di *et al*.^24^ Consistent with the findings in a large study by Salis *et al*.^25^, longer CPB time was associated with in-hospital mortality in our study. Reduced CPB time in the MHCA + SACP group could be attributed to higher core temperature and shorter cooling and re-warming times with MHCA + SACP group.

Low body temperature is protective against vital visceral organs and the spinal cord^26^. In comparison to the brain, the spinal cord is less sensitive but may still be injured during aortic surgery if body temperature remains high^27^. However, an important caveat during DHCA, with or without SACP, is the shunt of blood flow from the spinal cord injury to low-resistance vascular beds^28^. In the current study, no apparent spinal cord injury was observed in either group, suggesting that 22–25 °C is appropriate. An animal experiment reported that moderate hypothermia (20 °C) provided sufficient cerebral metabolic suppression, earlier recovery of electroencephalogram activity, and significantly downregulated expression of heat shock proteins (HSP-72)^29^. All together, these findings encourage the use of hypothermic instead of normothermic SACP.

Both under-perfusion and over-perfusion impair neurologic function after SACP. The choice of SACP parameters should be based on the brain temperature and the corresponding cerebral blood flow (CBF). Although maintenance of adequate CBF and cerebral metabolism (CMRO_2_) is critical during hypothermic circulatory arrest, the modulation of CBF/CMRO_2_ is very complex. In addition to brain temperature, CBF/CMRO_2_ could be affected by a variety of factors, including PaCO_2_, PaO_2_, blood viscosity, mean arterial pressure beyond the autoregulatory range, intracranial pressure and central venous pressure, pharmacologic agents, preoperative clinical conditions, CBF and flow-metabolism coupling, acid-base management and hemodilution. In a previous study, CPB surgery under moderate hypothermia was associated with low CBF (10–20 ml/100 g/min) and CMRO_2_ of 0.5 ml/100 g/min^30^, suggesting that SACP flow at 25–30 ml/min is safe and adequate for pediatric patients less than one year of age.

The current study has a number of limitations. First, the study was retrospective and the number of cases in both groups was relatively small to fully support statistical analysis. Also, the rate of complications other than in the central nervous system seems to be slightly higher in MHCA + SACP group, although the differences are not statistically different. Second, long-term outcomes were not assessed. Third, the complications were defined primarily according to clinical features, and not based on neurologic monitoring^31^. Large-scale, prospective, randomized controlled trials are needed to verify the preliminary findings of the current study.

To summarize, in comparison to DHCA alone, SACP in combination with MHCA is associated with less serious complication in pediatric patients receiving surgery for complex congenital aortic malformations under CPB.

## Methods

### Data collection

The study protocol was approved by the Ethics Committee of Children’s Hospital of Chongqing Medical University, China. We collected and analyzed data from patients who underwent one-stage corrective surgery for CoA/IAA with CPB at the Children’s Hospital of Chongqing Medical University, China, between January 2008 and December 2017. The patients were divided into two groups, a DHCA group and a MHCA + SACP group, based on the CPB method used. Hypothermia was categorized based on the recent Consensus of International Aortic Arch Surgery Study Group^32^: profound, ≤14 °C; deep, 14.1–20.0 °C; moderate, 20.1–28.0 °C; mild, >28 °C. DHCA was used more often in the earlier years of the study period whereas MHCA + SACP was used more often after the year 2012. We only analyzed data from patients whose CoA or IAA surgeries were performed by the same team of surgeons to minimize selection bias. The exclusion criteria included preoperative nervous system dysfunction, renal insufficiency and coagulopathy. Because this was a retrospective study, the need for informed consent was waived by the Ethics Committee of the Children’s Hospital of Chongqing Medical University.

### Anesthesia

Peripheral intravenous access was obtained 30 minutes before anesthesia induction with intravenous midazolam (0.1 mg/kg), penehyclidine hydrochloride (0.01 mg/kg), sufentanil (0.0015 mg/kg) and cisatracurium (0.1–0.15 mg/kg). None of the children were premedicated before the surgery. After tracheal intubation, anesthesia was maintained by intermittent inhalation of 1–2% sevoflurane via tracheal intubation. The inspired oxygen concentration was 30–50%, the tidal volume was 8–10 ml/kg, the respiratory rate was 20–28 breaths/min and the ratio of inspiration/expiration was 1:1.5. The right internal jugular vein, right radial artery and femoral artery were catheterized to monitor central venous pressure and arterial blood pressure in the upper and lower extremities. Throughout the operation, patients received continuous intravenous infusions of sufentanil (2–4 µg/kg/h) and cisatracurium (0.1–0.15 mg/kg/h).

### CPB procedures

CPB was conducted using a Stockert-C/III CPB machine (Germany), a Dideco 901/902 membrane oxygenator (Italy), and an arterial microembolism filter (Ningbo Fly Medical Healthcare Co., Ltd, China). Based on preoperative hemoglobin level and hematocrit, extracorporeal circuits were primed with irradiated and leukocyte-depleted red blood cells, fresh frozen plasma, bicarbonates, heparin and 20% albumin to 30% hematocrit. Methylprednisolone (30 mg/kg), creatine phosphate (1 g), furosemide (0.5 mg/kg) and ulinastatin (10000 IU/kg) were used routinely. The ascending aorta and superior and inferior vena cava were cannulated to establish CPB.

For children with IAA or preductal CoA combined with large ductus arteriosus, the pulmonary artery was cannulated through the ductus arteriosus into the descending aorta to ensure lower body perfusion. After the establishment of CPB, patients were cooled to the target temperature. When the nasopharyngeal temperature reached 32 °C, the ascending aorta was clamped for infusion with cardioplegic solution. For DHCA, circulation was arrested at a nasopharyngeal temperature of 18–20 °C. An ice cap was placed on the head of the patient during the period of cooling and circulatory arrest. Systemic circulation was restarted immediately upon the completion of aortic surgery. For MHCA + SACP, the aorta was cannulated through the ascending aorta into the innominate artery when nasopharyngeal temperature reached 25 °C. The SACP flow was 25–40 ml/min. The radial-arterial blood pressure was maintained at 25–40 mmHg (3.3–5.3 kPa) to ensure jugular venous oxygen saturation at 60% or greater. When the aortic arch surgery was complete, the aortic artery cannula was returned to the ascending aorta. After the air was fully pumped out, systemic perfusion was restarted. Rewarming started when venous oxygen saturation reached ≥65%. Other cardiac malformations, if present, were corrected in either the cooling or rewarming stage. Hematocrit was maintained at 25–30% throughout the CPB. Alpha-stat was used to manage blood gas. Zero-balanced ultrafiltration and modified ultrafiltration were performed in all patients.

### Coagulation management

ACT was monitored closely during the operation, especially in the preoperative period and after heparinization and upon neutralization. ACT was maintained at >480 s during CPB. Thrombelastogram (TEG) was used to guide appropriate management (fresh frozen plasma, platelets or cryoprecipitate) if significant bleeding was observed in the surgical field after neutralization.

### Sedation protocol

Children were sedated with 10–20 μg/kg/h midazolam or 4–6 mg/kg/h propofol. The choice was based on surgeon discretion.

### Perioperative data

Preoperative data included demographics (age and sex) and basic clinical features, including body weight, diagnosis, cardiac ejection fraction, serum creatinine, and blood urea nitrogen. Intraoperative CPB data included CPB time, aortic cross-clamping (ACC) time, DHCA or SACP time, lowest nasopharyngeal temperature, lowest rectal temperature and urine output during bypass. Postoperative data included 24-hour urine output, 24-hour chest drainage, inotropic score, time to regain consciousness, duration of mechanical ventilation and intensive care unit (ICU) stay. The following complications were assessed: (1) neurologic complications, including delayed recovery of consciousness, convulsion, coma, abnormal sensation, CT/MRI examination for brain edema and cerebral embolism, and spinal cord injury (hemiplegia and paraplegia); (2) acute renal failure (the need for dialysis); (3) low cardiac output syndrome; (4) acute respiratory distress syndrome; (5) death before discharge.

### Statistical analysis

We used SPSS 26.0 statistical software for statistical analyses. Normally distributed continuous variables are expressed as mean ± standard deviation, and analyzed with Student’s *t*-test. Continuous variables with skewed distributions are expressed as median with interquartile range, and analyzed with Mann-Whitney U test. Categorical variables were analyzed using chi-squared test or Fisher’s exact test as appropriate. Risk factors for in-hospital mortality were tested using multivariate logistic regression analysis. p < 0.05 is considered statistically significant.

### Consent to publish

All authors provided consent for publication without identifying patient information.

## Data Availability

The data sets generated and/or analyzed for the current study are available on request.
